# Evaluating a Co-Generational Program: Building Social Cohesion Across Generations

**DOI:** 10.1093/geroni/igaf122.3927

**Published:** 2025-12-31

**Authors:** BoRin Kim, Sofia Holmes, Kelley Petralia, Laura Cerniglia, Tracy LaCroix, Chung Hyeon Jeong, Alexa Johnson

**Affiliations:** University of New Hampshire, University of New Hampshire, New Hampshire, United States; University of New Hampshire, Durham, New Hampshire, United States; Westborough Connects, Westborough, Massachusetts, United States; Westborough Connects, Westborough, Massachusetts, United States; Westborough Connects, Westborough, Massachusetts, United States; University of New Hampshire - eHealth readiness, Durham, New Hampshire, United States; University of New Hampshire, Durham, New Hampshire, United States

## Abstract

Meaningful intergenerational interactions support both older adults’ well-being and adolescent development, yet declining opportunities for engagement contribute to widening social divides in communities. Intergenerational solidarity theory offers a framework for understanding how structured cross-generational contact fosters affective, associational, and functional ties that strengthen cohesion and belonging. Guided by this framework and developed in collaboration with a local community organization, this study evaluated a dyadic co-generational program designed to promote reciprocal connection and civic engagement. We examined how participation influenced social cohesion, perceived connections, and community belonging among older adults and adolescents. Eleven intergenerational pairs of older adults (65+) and adolescents (14–17) participated in a six-week program in which dyads selected and completed weekly community-based activities of their choice. Data were collected through semi-structured post-program group interviews and weekly activity logs, enabling triangulation across self-reported experiences and observed practices. Thematic analysis identified mechanisms of connection, perceived benefits, and barriers to sustained engagement. Shared activities emerged as the primary mechanism fostering connection and reciprocity. Reported benefits included reduced social isolation, stronger community belonging, and more positive perceptions of both age groups. Older adults emphasized the program’s role in countering isolation in age-segregated housing, while adolescents valued opportunities to engage with the broader community beyond peers. Barriers included transportation, communication differences, and informal power dynamics. Findings highlight the importance of designing programs collaboratively with community partners to enhance feasibility, cultural fit, and long-term impact. Insights from this program advance understanding of intergenerational solidarity while offering practice-based strategies to foster more inclusive, connected communities.

